# *Agricultural and Food Economics*: the challenge of sustainability

**DOI:** 10.1186/s40100-020-00156-2

**Published:** 2020-04-24

**Authors:** Gianluca Brunori, Giacomo Branca, Luigi Cembalo, Marijke D’Haese, Liesbeth Dries

**Affiliations:** 1 Department of Agriculture, Food and Environment, University of Pisa, via del Borghetto 80, 56124 Pisa, Italy; 2 Department of Economics, Engineering, Society and Business Organization – DEIM, Tuscia University, Via Santa Maria in Gradi, 4, 01100 Viterbo, VT Italy; 3 Department of Agricultural Economics and Policy, University of Naples “Federico II”, Corso Umberto I, 40, 80138 Napoli, NA Italy; 4 Department of Agricultural Economics, Ghent University, St. Pietersnieuwstraat 33, 9000 Gent, Belgium; 5 Agricultural Economics and Rural Policy Group, Wageningen University, 6706 KN Wageningen, The Netherlands

The Agenda 2030 of the United Nations and the Paris climate agreement, both signed in 2015, have given a new frame of reference to the international scientific community. Food is at the center of this debate. The question of ‘How to feed the world’ in the context of a growing world population—Sustainable Development Goal n. 2, ‘zero hunger’—requires revisiting in the light of a growing urbanization and of the multiple dimensions of nutrition. In addition, the current coronavirus pandemic highlights the vulnerability of our global food supply systems to disruptions leading to risks for consumers’ safety, shortages in labor, and other factors of production as well as in internationally traded foodstuffs.

Existing food systems contribute to malnutrition, resource depletion, food waste, pollution, concentration of power, and generation of inequalities. At the same time, they are threatened by climate change. A growing number of recent top-level science-policy bodies, such as the UN World Committee for Food Security, the IPCC, the EAT-Lancet commission, the UNDP, and the IPES-food have sent a similar message: business as usual is not an option, a radical change is needed. The necessary changes are the following: consumption patterns should be aligned with healthy and sustainable diets, production systems should reduce their pressure on natural resources, food supply systems in both developed and emerging economies should be made more resilient, and effective enforcement, monitoring, and evaluation systems for policies should be developed. Technological, social, and policy innovations should be mobilized to support this effort.

The emphasis on societal challenges has important implications for economists, as it implies rethinking the roles of market forces, of businesses and of public policies. The business world is already reflecting on this, as the recent declaration of the US Business Roundtable Group on the primacy of social responsibility on profits shows. As for market forces, the reflection on trade policies, on investment criteria, and on competition policies has started.

Food is largely recognized as a testbed for system innovation, given the role of natural cycles in food-related activities. For example, in Europe, the ambitious Green Deal of Van der Leyen includes a Biodiversity strategy, a “Farm to Fork” strategy, a Zero Pollution goal, and a Circular Economy action plan. Domestic and international agricultural policies should also undergo a new wave of reforms aligned with these goals, and a growing number of voices claim that an effective food policy should be designed to better deliver on these ambitions.

However, new policies cannot be built on old knowledge. For agricultural and food studies, the new context and demand for policy reforms encourage the scientific community to add new topics to the research agenda. The understanding of sustainability as a set of goals based on trade-offs between economic, social, and ecological dimensions entails new paradigms, theories, and methods. We propose here four cross-cutting areas of reflection for our authors and readers:

- System approaches, addressing the relations between activities, actors, and outcomes related to food and their evolving patterns in changing environments
- Nexus approaches, addressing the impact that intervention in one sector—for example food—could generate on other sectors—for example health, water, energy, and land
- Future-oriented approaches, not taking for granted that past trends will determine future events, and paying attention to unintended consequences of choices at all levels, from consumption to policy to technology, and to the adaptation needs
- Inter- and trans-disciplinary approaches, implying the enlargement of the areas of overlap and synergies with other disciplines.

Our journal aims at encouraging a reflection on these aspects, with the aim to give a small but hopefully significant contribution to the collective effort to achieve the Sustainable Development Goals.

